# Spontaneous hematoma of the rectus sheath following percutaneous transluminal coronary angioplasty and stent placement

**DOI:** 10.11604/pamj.2018.30.88.15000

**Published:** 2018-05-30

**Authors:** Rodolfo Mendes Queiroz, Fred Bernardes Filho

**Affiliations:** 1 Department of Radiology and Imaging, Santa Casa da Misericórdia of Avaré, Avaré, São Paulo, Brazil; 2 CENTROMED Diagnóstico por Imagem, Avaré, São Paulo, Brazil; 3 Dermatology Division, Department of Medical Clinics, Ribeirão Preto Medical School, University of São Paulo, Ribeirão Preto, Brazil

**Keywords:** Hematoma, abdominal pain, percutaneous transluminal coronary angioplasty

## Image en médecine

A 74-year-old female with hypertension, coronary artery disease, was hospitalized due to an acute myocardial infarction. A percutaneous transluminal coronary angioplasty (PTCA) and stenting were performed successfully. After six hours of PTCA, an intense abdominal pain localized on right iliac fossa started. On physical examination, a palpable, painful, firm, nonpulsatile abdominal mass was observed. Fothergill’s and Carnett’s signs were present; there were not Cullen's and Grey Turner's signs. Abdominal computed tomography (CT) showed oval collection, isodense in relation to the muscular planes, located in almost all the extension of the rectus abdominis muscle on the right, measuring in its greater axial axes 8.6 x 6.0 cm. Patient did not present drop of the hemoglobin levels nor hemodynamic instability. The diagnosis of hematoma of the rectus sheath was performed and conservative management was elected. Rectus sheath hematoma (RSH) is an uncommon cause of acute abdominal pain and can be life threatening if diagnosis and management is delayed. RSH occurs due to the accumulation of blood within the rectus abdominis sheath secondary to bleeding from damaged inferior or superior epigastric arteries, or through direct tears of the rectus abdominis muscle. Abdominal pain and fall in hemoglobin with presence of risk factors (especially systemic anticoagulation drugs like warfarin and intravenous heparin) constitute important clinical clues to the diagnosis. Management is guided by the size of the haematoma and haemodynamic stability of the patient. Clinicians should consider RSH in the differential diagnosis of acute abdominal pain.

**Figure 1:**
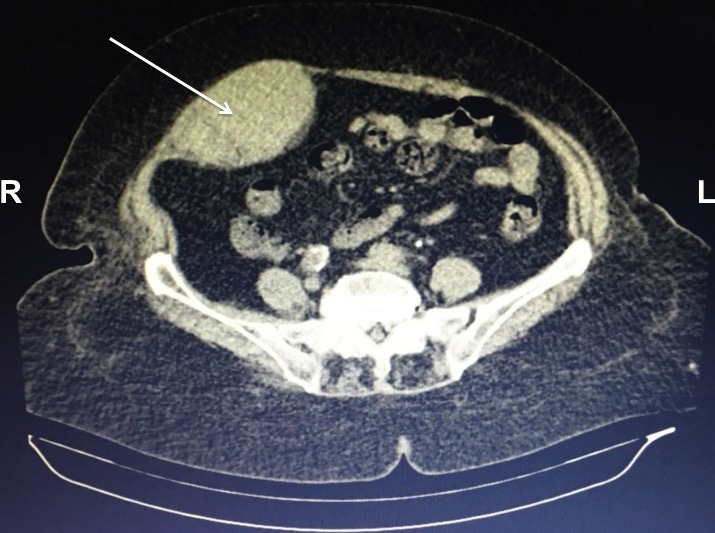
Computed tomography of abdomen showing hematoma (arrow) within rectus sheath; L: left; R: right

